# 1/*f* Noise and Machine Intelligence in a Nonlinear Dopant Atom Network

**DOI:** 10.1002/smsc.202000014

**Published:** 2021-01-15

**Authors:** Tao Chen, Peter A. Bobbert, Wilfred G. van der Wiel

**Affiliations:** ^1^ NanoElectronics Group MESA+ Institute for Nanotechnology and BRAINS Center for Brain-Inspired Nano Systems University of Twente PO Box 217 Enschede AE 7500 The Netherlands; ^2^ Molecular Materials and Nanosystems & Center for Computational Energy Research Department of Applied Physics Eindhoven University of Technology PO Box 513 Eindhoven MB 5600 The Netherlands

**Keywords:** 1/*f* noise, brain, criticality, intelligence, network, nonlinearity

## Abstract

Noise exists in nearly all physical systems ranging from simple electronic devices such as transistors to complex systems such as neural networks. To understand a system's behavior, it is vital to know the origin of the noise and its characteristics. Recently, it was shown that the nonlinear electronic properties of a disordered dopant atom network in silicon can be exploited for efficiently executing classification tasks through “material learning.” Here, we study the dopant network's intrinsic 1/*f* noise arising from Coulomb interactions, and its impact on the features that determine its computational abilities, viz., the nonlinearity and the signal‐to‐noise ratio (SNR), is investigated. The findings on optimal SNR and nonlinear transformation of data by this nonlinear network provide a guideline for the scaling of physical learning machines and shed light on neuroscience from a new perspective.

## Graphical abstract

1/*f* noise is found to be fundamentally coupled with the machine intelligence of a dopant network in silicon. The network's 1/*f* noise and machine intelligence both originate from its internal nonlinear interactions. Rather than attempting to minimize the noise, as in traditional electronics, the dopant network is balanced between its signal‐to‐noise‐ratio and information processing capability, at optimal bias conditions.

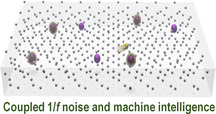

## 1 Introduction

In 1/*f* noise, the power spectral density (PSD) of the noise is inversely proportional to the frequency *f*. A 1/*f* PSD reflects the scale‐invariant correlations of the underlying physical processes in a broad scope of systems for information processing.^[^ ^1^, ^2^, ^3^, ^4^, ^5^, ^6^, ^7^ ^]^ The slow fluctuations corresponding to the low‐frequency end of 1/*f* noise impose boundary conditions on the systems’ operation, which sometimes require additional signal conditioning techniques such as filtering. Therefore, understanding the underlying mechanism(s) of 1/*f* noise facilitates achieving optimal performance, by optimizing the individual components and the system design. In doped semiconductors, the 1/*f* noise was attributed to electron trapping and de‐trapping,^[^ ^8^ ^]^ whereas recently Burin et al. suggested that this noise involves transitions of multi‐electron clusters between two almost degenerate states.^[^ ^9^ ^]^ Healthy brains also exhibit 1/*f* noise,^[^ ^6^, ^7^ ^]^ hypothetically because the large‐scale complex neural networks are poised at criticality,^[^ ^6^, ^10^, ^11^, ^12^ ^]^ i.e., at the border of a phase transition such as the onset of synchronous activity.^[^ ^4^, ^12^ ^]^ In spite of the occurrence of 1/*f* noise in many natural and artificial systems, and decades of research, a unified explanation of 1/*f* noise has not been agreed upon. In the context of developing efficient physical hardware for machine intelligence,^[^ ^3^, ^5^, ^13^, ^14^, ^15^, ^16^ ^]^ 1/*f* noise in individual components, such as memristors, has been studied.^[^ ^3^, ^5^ ^]^ Yet, the mechanisms underlying 1/*f* noise may differ in different electronic devices^[^ ^1^, ^3^, ^9^, ^17^ ^]^ and vary across scales from device level to system level. Whether there is a correlation between the presence of 1/*f* noise in a complex network and its capability of information processing is an open question.^[^ ^18^, ^19^ ^]^ In large‐scale biological or man‐made hardware networks,^[^ ^6^, ^10^, ^11^, ^12^, ^14^ ^]^ the computational capability stems from complex nonlinear interactions, which can, at the same time, lead to critical behavior and, therefore, 1/*f* noise.^[^ ^12^ ^]^ That is to say, nonlinear interactions may be a two‐edged sword. So far, how to achieve the optimal computational properties of large‐scale physical networks in the presence of emergent 1/*f* noise has remained elusive. Here, we examine the 1/*f* noise of a dopant atom network in silicon in the variable‐range hopping (VRH) regime.

The dopant network,^[^ ^14^ ^]^ formed by electrostatically coupled dopant atoms in silicon and referred to as dopant network processing unit (DNPU),^[^ ^20^, ^21^ ^]^ has a typical footprint of only 300 × 300 nm^2^ and consumes a power of ≈1 μW or even less. We have shown that a single DNPU is capable of carrying out canonical machine learning tasks using “material learning” techniques.^[^ ^13^, ^14^ ^]^ From a dopant network connected to eight electrodes, we can choose *M* input electrodes on which voltages representing input data are applied and one output electrode where output current is measured. Then, the input–output relation can be configured by the voltages applied on the remaining 7 − *M* control electrodes. Through artificial evolution of the control voltages by a genetic algorithm, thereby tuning the potential landscape, the intrinsic nonlinearity of the DNPU can be harnessed for information processing. We previously demonstrated that the DNPU can perform a range of nonlinear classification tasks, such as arbitrary Boolean logic in a 2‐input‐1‐output (*M* = 2) configuration and image feature filtering in a 4‐input‐1‐output configuration (*M* = 4). The DNPU leverages the atomic‐scale interactions among localized dopant states for computation, thus potentially achieving unprecedented energy efficiency and computational density comparable to the human brain.^[^ ^13^, ^14^ ^]^ The DNPU can be an efficient building block for machine intelligence.^[^ ^14^ ^]^ Understanding the effect of 1/*f* noise is, therefore, crucial for scaling up DNPU‐based learning machines.^[^ ^20^, ^21^ ^]^

Through electrical measurements, we show here that the 1/*f* noise power and the DNPU response to external signals scale differently with the mean output current. As a consequence, the DNPU's signal‐to‐noise ratio (SNR), related to its dynamic range, shows a peak when plotted against the bias voltage that energizes the hopping transport in a three‐terminal measurement. The DNPU's computational capability, which we ascribe to its nonlinearity,^[^ ^13^, ^14^, ^15^, ^16^ ^]^ diminishes with rising SNR. Our results suggest that a DNPU should be biased at a critical point to enable a discernable response to an input signal on the one hand and to retain the ability of nonlinear data transformation on the other hand. We deem this an important guiding principle for natural computing^[^ ^5^, ^13^, ^14^, ^15^, ^16^ ^]^ and speculate that the dependence of performance on bias conditions can be generalized to biological neural networks and potentially facilitates the understanding of the functioning of brain.^[^ ^22^, ^23^, ^24^, ^25^, ^26^, ^27^ ^]^

## 2 1/*f* Noise in Hopping Conduction

**Figure 1:**
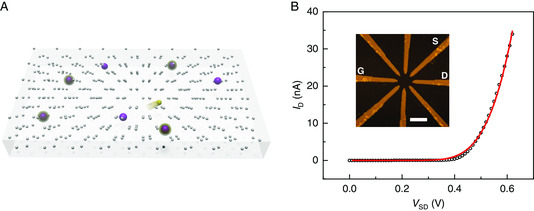
Hopping conduction through a dopant network. A) Schematic of charge carrier (yellow) hopping among dopant atoms (purple spheres) in silicon (grey spheres). B) Drain current (DC) at 77 K as a function of voltage between source (S) and drain (D), with grounded drain and gate (G). The red solid line is a fit with a model for voltage‐activated hopping conduction.^[^ ^14^ ^]^ The inset shows an atomic force microscope image of the dopant network device, which consists of doped silicon (dark region) and eight nanoelectrodes. The scale bar is 300 nm.

In the hopping regime, the charge carriers, holes in boron‐doped silicon (or electrons in arsenic‐doped silicon, see Supporting Information), hop sequentially from one dopant atom to another when a bias voltage is applied (**Figure** **1**A). The hopping rate decays exponentially with distance and energy difference between two hopping sites, when this energy difference is positive.^[^ ^28^ ^]^ Together with the electrostatic Coulomb interactions between all charges, this leads to nontrivial electronic properties. As a result, the hopping conduction through the dopant network exhibits complex nonlinear behavior.^[^ ^14^ ^]^ This nonlinearity results in intricate dynamics and is a useful asset for information processing. We have previously observed that the output current of the network exhibits fluctuations that are intrinsic to the device.^[^ ^13^, ^14^ ^]^ We will address these fluctuations, or noise, in the present work. To quantify the DNPU's noise, we performed current–voltage (*I–V*) measurements by applying a bias voltage to a source electrode (see the inset of Figure 1B) and measuring the current output at an adjacent drain electrode (essentially grounded by the *I*/*V* converter). One of the other electrodes, the gate electrode, was grounded, and the remaining electrodes were floating. The main panel of Figure 1B shows the mean drain current (DC) versus bias voltage, which clearly exhibits nonlinearity and can be modeled as voltage‐activated hopping conduction.^[^ ^14^ ^]^

**Figure 2:**
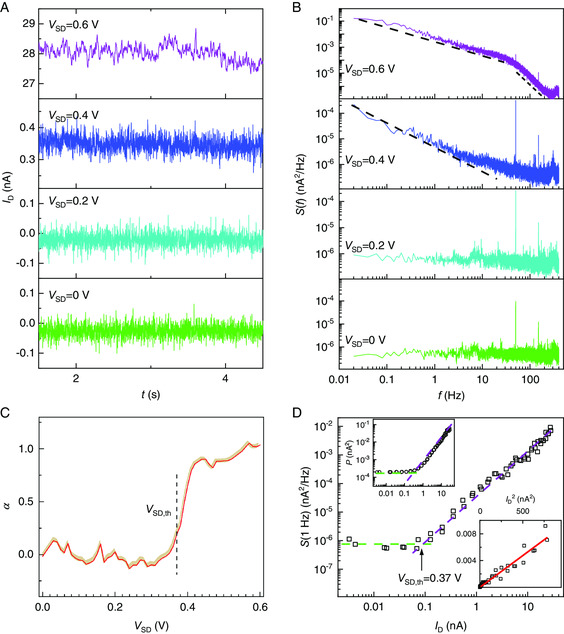
1/*f* noise in the hopping conduction regime. A) DC traces at different bias voltages, shown for 3 s segments (see Figure S1, Supporting Information, for segments of 500 s). B) PSD of the current traces. The dashed lines in the top two panels are guides to the eye, corresponding to *S *∝ 1/*f*. The short‐dashed line in the top panel indicates the roll‐off of the low‐pass filter of the measurement setup (≈70 Hz, see Experimental Section and Figure S2, Supporting Information). The peaks at 50 and 150 Hz are due to interference from the mains source. C) The exponent *α* as obtained by fitting the PSDs at different bias voltages as shown in (B) to $S(f)\propto 1/f^{\alpha }$. The yellow shade around the red curve shows the 95% confidence interval of the fit. We note that this interval indicates the good fitting quality, but not the standard deviation of *α* upon repeated sampling. At low bias, *α* fluctuates around zero, indicating white noise. When the bias voltage exceeds the threshold *V*_SD,th_ (black dashed line) defined in (D), *α* increases and eventually settles around 1, indicating 1/*f* noise. D) Noise power *S*(*f* = 1 Hz) plotted against the DC on a logarithmic scale. The green and purple dashed lines fit in the two different noise regimes. They intersect around 0.08 nA, corresponding to a bias voltage of around 0.37 V. The lower inset shows *S*(1 Hz) as a function of the square of the DC, and the red curve is a linear fit. According to Hooge's law,^[^ ^9^, ^17^ ^]^ the noise PSD 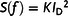. Here, we use, for simplicity, one proportionality constant *K* to encompass a few parameters. The upper inset shows the total noise power integrated over the whole frequency range (400 Hz) versus the DC. As in the main panel, two regimes are identified.

Four typical current traces are plotted in **Figure** **2**A. Their corresponding noise PSDs *S*(*f*) (see Experimental Section) are plotted in Figure 2B. At low bias voltages, the current traces show the characteristics of white noise (Figure 2A, lowest panel), as confirmed by the flat PSD (Figure 2B, lowest panel). This white noise originates from the measurement setup, mainly from the input resistance of the *I*/*V* converter (102 kΩ, see Experimental Section), and is independent of the device under study. Low‐frequency noise emerges at larger bias voltages (from 0.4 V onward).

The PSDs in Figure 2B (top two panels) follow a power law $S(f)\propto 1/f^{\alpha }$. Theoretically, *α* equals 1 in the VRH regime.^[^ ^9^ ^]^ We extracted the corresponding exponents *α* by fitting the PSDs on a logarithmic scale. When the bias voltage exceeds a threshold voltage *V*_SD,th_ (defined below), the exponent *α* increases from zero to a value in the range between 0.8 and 1 (Figure 2C). In three datasets collected from two independent devices (one boron DNPU, one arsenic DNPU), the exponents fall in similar ranges without significant bias‐dependence above *V*_SD,th_ (see Figure S3 and S4, Supporting Information). This range of exponents agrees with previous reports of 1/*f* noise arising from hopping conduction in the impurity band of doped silicon.^[^ ^2^ ^]^ We plot the PSD at 1 Hz *S*(1 Hz) as a function of the DC in Figure 2D, where two regimes are visible. When the current exceeds ≈0.08 nA, the noise power is proportional to the current squared, in accordance with Hooge's law (Figure 2D, lower inset).^[^ ^9^, ^17^ ^]^ Below ≈0.08 nA, the noise intensity does not change with current, corresponding to the noise floor of the measurement equipment. The intersection of the straight lines fitting these two trends is defined as the threshold voltage *V*_SD,th_ (black arrow in Figure 2D), which marks the transition to hopping‐dominated noise. The total noise power, obtained by integrating the PSD over the full bandwidth, also shows two regimes (Figure 2D, upper inset). The intersection of the noise power regimes occurs at a larger voltage than *V*_SD,th_ defined for *S*(1 Hz), because with increasing bias, the low‐frequency noise exceeds the noise floor earlier than the higher frequency noise.

In VRH conduction, a single hop of an electron or hole not only alters the occupation of the source and destination dopant sites,^[^ ^9^ ^]^ but also influences the potential energies of other dopant atoms in the whole network because of the Coulomb interaction. The change in potential landscape causes rearrangements of clusters of charge carriers at different time scales.^[^ ^9^ ^]^ The collective rearrangement of larger clusters features a larger time constant, associated with a lower frequency, and induces larger fluctuations of the network's conductivity than small clusters, leading to the characteristic 1/*f* noise.^[^ ^9^ ^]^ As the DNPU needs to be in the VRH regime to function,^[^ ^14^ ^]^ 1/*f* noise is concomitant with computational functionality. Therefore, 1/*f* noise in DNPU plays a different role from in conventional electronic devices that normally operate in the band‐conduction regime.

## 3 SNR under External Stimulation

**Figure 3:**
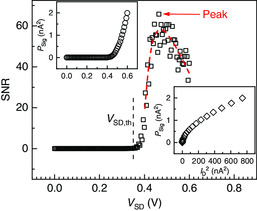
SNR as a function of source–drain voltage *V*_SD_. As the bias voltage increases, the SNR first increases to a peak located around 0.45 V, and then decreases (red dashed curve is a guide to the eye). Upper inset: the measured signal power (see Experimental Section) due to the gate modulation of 0.1 V and 1 Hz. The signal rises when the bias voltage crosses threshold *V*_SD,th_ (defined in Figure 2D). Lower inset: the signal power plotted as a function of the squared DC for easy comparison with the noise scaling (Figure 2D, lower inset), showing a sublinear dependence.

To investigate how the DNPU responds to external stimulation, we applied a small sinusoidal voltage signal (0.1 V amplitude, 1 Hz frequency, see Experimental Section and Supporting Information) to the gate electrode (Figure 1B, inset) and recorded the DC for different bias voltages applied to the source.^[^ ^14^ ^]^ The gate electrode is far away from the source and drain electrodes. Therefore, the current between the gate electrode and the source/drain electrodes is below the noise floor and, thus, negligible, as confirmed by *I*–*V* measurements. The resulting 1 Hz signal superimposed on the DC has been extracted with a lock‐in amplifier using the gate voltage as a reference (see Experimental Section). A 1 Hz signal emerges when the bias voltage crosses the threshold *V*_SD,th_ (**Figure** **3**, upper inset). The SNR, defined as the ratio of the output signal power at 1 Hz to the total noise power (see Experimental Section), exhibits a peak around a source−drain voltage of 0.45 V (Figure 3). This peak results from the different scaling of signal and noise with the DC. The noise power scales linearly with DC squared according to Hooge's law (Figure 2D, lower inset), whereas the signal scales in a sublinear way (Figure 3, lower inset). The sublinear dependency of the signal on DC can be understood as follows. When the bias voltage increases, the dopant energy level shifts induced by the 0.1 V gate modulation become relatively weaker, and therefore, the corresponding output signal superimposed on the DC also becomes relatively weaker (see Supporting Information for an analytical formulation).

The SNR peak observed here differs from what is observed in the so‐called stochastic resonance experiments in living systems.^[^ ^23^, ^24^, ^25^ ^]^ In such experiments, *external* (white) noise is added to a weak time‐dependent input signal. The SNR then maximizes at a certain noise intensity, a mechanism also known as noise‐induced threshold crossing.^[^ ^29^ ^]^ Our present study, however, implies that the DNPU's response to an external signal maximizes when the system is energized (voltage biased) optimally with respect to its *internal* (1/*f*) noise. On the one hand, the network must be sufficiently energized to allow for a measurable response signal. On the other hand, the bias voltage should not be too large, to not overshadow the effects of the external stimulation.

## 4 Nonlinearity and Machine Intelligence

**Figure 4:**
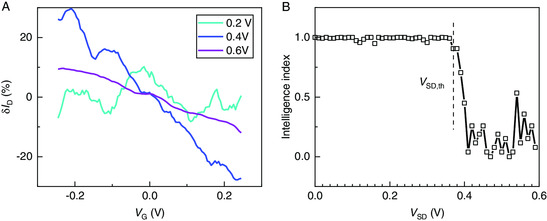
Nonlinearity and intelligence index of the dopant network. A) Percental DC change δ*I*_D_ as a function of the gate voltage for different source–drain biases. The gate voltage increases from −0.25 to 0.25 V in around 16 s. The current was smoothed with the Savitzky–Golay method (see Experimental Section). At low source–drain bias, e.g., 0.2 V, the current depends nonmonotonically on the gate voltage. It becomes more monotonic at larger bias voltages. B) The intelligence index calculated according to Equation (1) falls as the bias voltage exceeds the threshold (defined in Figure 2D), due to the diminishing nonlinearity in (A).

To characterize the DNPU's capability to process information at different bias voltages, we slowly ramped up the gate voltage from −0.25 to 0.25 V (see Experimental Section) and acquired a complex gate effect on the DC.^[^ ^14^ ^]^ With a low bias voltage applied to the source electrode, the gate voltage modulates the output DC in a highly nonlinear way (**Figure** **4**A), displaying nonmonotonic features and leading to both negative and positive transconductance, *G*_mn_ and *G*_mp_. This behavior resembles the inhibition and excitation of biological neurons,^[^ ^22^ ^]^ enabling not only performing additive operations but also operations that need negation such as NAND and NOR Boolean functions. As the bias voltage increases, the nonlinearity is reduced, and the current changes monotonically under gate modulation. For a large bias voltage, the charge carriers always hop along the resulting strong source–drain electric field,^[^ ^30^ ^]^ so that the gate‐induced electric field is not able to significantly affect the current.

The computational power of our dopant network device^[^ ^13^, ^14^ ^]^ as well as that of other physical computing hardware^[^ ^15^, ^16^ ^]^ is largely attributed to their nonlinearity. A suitable nonlinear transformation of linearly inseparable input data into a high‐dimensional space can make the data linearly separable, thus facilitating classification.^[^ ^31^ ^]^ Therefore, we define a simple “intelligence index”

(1) $$1-({\left(\left(n\left(G_{\text{mp}}\right)-n\left(G_{\text{mn}}\right)\right)/\left(n\left(G_{\text{mp}}\right)+n\left(G_{\text{mn}}\right)\right)\right)}^{2}$$

based on the nonlinearity of the curves in Figure 4A, to assess the potential computational capacity, where *n* is the number of data points with positive or negative transconductance. The intelligence index reaches 1 when there are equal numbers of positive and negative transconductance points, and drops to 0 when the current changes monotonically with gate voltage. It requires both additive and subtractive operations to perform nonlinear classification tasks such as the prototypical linearly inseparable XOR problem,^[^ ^13^, ^14^ ^]^ as is evident by the expression *A* XOR *B* = *A* + *B* − 2*AB*, where *A* and *B* are two inputs that can be either 0 or 1. Therefore, the existence of both positive and negative transconductance, i.e., a non‐zero intelligence index, is favorable and a necessary condition for the network to perform nonlinear classification tasks. As most real‐life classification tasks are nonlinear, we consider the intelligence index a useful indicator of the DNPU's potential computational capability, even though the exact functionalities also rely on other criterions such as dopant concentration and electrode configuration. As displayed in Figure 4B, the intelligence index starts to fall when the bias voltage crosses the threshold *V*_SD,th_ (Figure 4B), which holds for different sampling frequencies (see Experimental Section). The rising edge of the SNR, thus, coincides with the falling edge of the intelligence index, suggesting that the network should be energized near *V*_SD,th_ for optimal information processing. This observation (see also Figure S3 and S4, Supporting Information) is intriguing and remarkably consistent with our previous findings from an exhaustive search for computational functionality,^[^ ^14^ ^]^ which has revealed that there exist optimal ranges of control voltages energizing the dopant network.

The observations reported earlier are established in both boron and arsenic DNPUs (see Figure S3 and S4, Supporting Information). We speculate that a peak in the SNR, as well as a co‐occurrence of rising SNR and diminishing nonlinearity, exists in similar nonlinear networks when their bias conditions are changed. The activation of a biological neural population is also a nonlinear function (as in Figure 1B) of its total energizing input current,^[^ ^22^ ^]^ and the 1/*f* noise is found to be concomitant with neural activity.^[^ ^6^, ^7^ ^]^ We propose that neurological experiments are carried out to validate this speculation. These experiments will enhance the understanding of neural modulation and potentially advance brain‐disorder treatments, such as deep‐brain stimulation techniques.^[^ ^26^, ^27^ ^]^ It is worth mentioning that the properties of the DNPU's electron dynamics, i.e., nonlinear interactions, many‐electron rearrangements, and a stationary response under steady‐state driving (Figure S1, Supporting Information), are sufficient conditions for so‐called self‐organized criticality (SOC),^[^ ^4^, ^32^ ^]^ which has been claimed to occur in the brain.^[^ ^6^, ^10^, ^12^ ^]^ In SOC, an avalanching system with nonlinear internal interactions can self‐organize to criticality without fine‐tuning of any control parameter.^[^ ^32^ ^]^ Bak et al. have proposed SOC as the underlying mechanism of 1/*f* noise.^[^ ^33^ ^]^ Although direct evidence for the occurrence of SOC in the dopant network, such as the observation of multi‐scale avalanching,^[^ ^34^ ^]^ is still lacking, it is worth noticing the common attributes, in particular, the 1/*f* noise in both dopant networks and neural networks.

## 5 Conclusion

As in conventional transistors, the electronic properties of DNPUs vary under different bias conditions. Yet, their large number of internal degrees of freedom exceeds that of conventional electronic components and gives rise to functionality comparable to that of small neural networks.^[^ ^14^, ^22^ ^]^ Unlike conventional electronics, the functionality and 1/*f* noise of DNPUs are fundamentally linked to their complexity and nonlinear behavior, which may also be the case in neural networks. Therefore, the conditions for optimal information processing by the dopant network in the presence of the concomitant 1/*f* noise^[^ ^35^ ^]^ can potentially be generalized to other complex nonlinear physical systems. These general rules are anticipated to increase our understanding of both artificial and natural intelligence. Concepts from neuroscience have largely stimulated the development of artificial intelligence.^[^ ^5^, ^12^, ^13^, ^14^, ^31^ ^]^ Now, reversely, concepts from physical systems exhibiting a rudimentary form of artificial intelligence may yield useful insights into the principles behind the working of the brain.

## 6 Experimental Section

6.1

6.1.1

##### Device Fabrication

We have used both boron and arsenic DNPUs in this study. The device fabrication is detailed in our previous work.^[^ ^14^ ^]^ For boron DNPU, the boron atoms have been implanted in an n‐type silicon substrate (resistivity 1–10 Ω cm), with a boron surface concentration exceeding 10^20^ atoms cm^−3^ to ensure ohmic contact with the electrodes. After patterning Pd/Ti nanoelectrodes, the central silicon area that is not covered by the electrodes has been etched back to reduce the dopant concentration to the order of 10^17^ atoms cm^−3^. The arsenic dopant network device has been fabricated following the same procedure, but with a p‐type silicon substrate (resistivity 1–20 Ω cm) and Al/Ti electrodes. Both types of devices can be evolved to perform arbitrary Boolean logic functions in the way described in our previous work.^[^ ^14^ ^]^

##### Measurement Setup

The dopant network devices were cooled down to 77 K with a customized dipstick in liquid nitrogen to enter the VRH regime.^[^ ^14^ ^]^ The DC voltages were applied by an electronics rack equipped with digital‐to‐analog converters (DACs), and the output current was converted to a voltage with an *I*/*V* converter. In the present study, the parasitic wire capacitances (≈4 nF) and input resistance (102 kΩ when the gain was set to 100 MΩ) of the *I*/*V* converter result in a bandwidth of about 200 Hz. Before digitizing, an isolated‐output module (set at 100 Hz bandwidth) following the *I*/*V* converter further limited the bandwidth of the output signal for anti‐aliasing purposes. The generation of the input waveform for the gate modulation and the data acquisition of the output current were implemented with an Adwin‐Gold II module, a real‐time waveform generator and digitizer. The sampling frequency *f*_s_ is 800 Hz for noise data collection and for sensitivity measurements. The noise traces were sampled for 500 s (Figure S1, Supporting Information).

For the nonlinearity characterization, the sampling frequency was reduced to 300 Hz. The gate voltage rises from −0.25 to 0.25 V with 0.1 mV per step. Further reducing the sampling frequency resulted in slightly different curves due to hysteresis, but qualitatively the same extent of nonlinearity, confirming the robustness of the proposed intelligence index.

To measure the setup's bandwidth, we performed a two‐terminal test. A sine wave (10 mV amplitude) with a frequency increasing from 1 to 200 Hz was superimposed on the bias voltage applied to the source electrode. The output signal at each corresponding frequency is extracted and plotted in Figure S2, Supporting Information. The roll‐off beginning at ≈70 Hz confirms that the slope at the high‐frequency end in the top panel of Figure 2B (see the short‐dashed line) is due to the measurement setup.

##### Signal Processing

The PSD of the output signal has been evaluated with Welch's method, i.e., by splitting up the output signal into overlapping shorter segments, and averaging their corresponding PSDs calculated with periodogram to reduce the fluctuations in the overall PSD.

To extract the signal superimposed on the output current at a specific frequency *f*, we used the principle of a lock‐in amplifier. The output current waveform vector is denoted by *I*_D_(*k*), where *k* runs from 1 to *N*, the total number of data points. The normalized inner product of the current waveform with sin(2*πkf*/*f*_s_) and cos(2*πkf*/*f*_s_) yields the in‐phase and quadrature components of the signal at *f*, respectively.

(2) $$X=2I_{D}(k)\;\sin(2\pi kf/f_{s})/N$$

(3) $$Y=2I_{D}(k)\;\cos(2\pi kf/f_{s})/N$$

The signal amplitude is then $I_{\text{sig}}=\sqrt{X^{2}+Y^{2}}$, and the signal power is 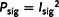. The phase is *θ *= arctan(*Y*/*X*). The phase *θ* corresponding to the dataset shown in Figure 3 is plotted against the bias voltage in Figure S5, Supporting Information. As shown, at low bias, the phase is around 80°, indicating a weak signal due to capacitive coupling between the wires of the measurement setup. As the bias voltage exceeds the threshold *V*_SD,th_, the phase drops to nearly zero. This drop implies that the quadrature component *Y* due to capacitive coupling is now negligible compared with the signal caused by the gate modulation. Theoretically, as long as the input signal frequency is much smaller than the intrinsic hopping rate of the dopant network (on the order of 100 MHz), the output signal amplitude should be the same (see also Supporting Information). However, in practice, we keep the input frequency low (1 Hz) to reduce cross coupling. Further decrease in the frequency, e.g., to 0.5 Hz, does not change the behavior of SNR.

To reveal the nonlinearity of the DC under gate modulation (Figure 4A), we adopt the Savitsky–Golay method, i.e., fitting a segment of the current trace (corresponding to 0.1 V interval of gate voltage) with the second‐order polynomials to estimate the current in the middle of the range, which filters the noise and smoothens the data.

## Conflict of Interest

The authors declare no conflict of interest.

## Supporting information

Supplementary Material
