# Supplementary material for: 1/f Noise and Machine Intelligence in a Nonlinear Dopant Atom Network
Source: Small Sci. 2021 Jan 15;1(3):2000014. doi: 10.1002/smsc.202000014 (PMC11935790; doi:10.1002/smsc.202000014)
Supplement: Supplementary file 1 — Supplementary Material [file SMSC-1-2000014-s001.docx]

Supporting Information

1/*f* noise and machine intelligence in a nonlinear dopant atom network

Tao Chen^1^, Peter A. Bobbert^1,2^, Wilfred G. van der Wiel^1^*

**Sublinear dependence of signal vs drain current:**

The dopant network device at 77 K operates in the 2D Mott-VRH regime^[14]^. The drain current *I*_D_ vs bias voltage *V*_SD_ can be modeled as voltage-activated variable-range hopping conduction following

$I_{D}=V_{\mathrm{SD}}G_{h}e^{-\left( \frac{{k_{B}T}_{h}}{qEr} \right)^{\frac{1}{3}}}$, (s1)

where *G*_h_ is a prefactor only weakly dependent on temperature, *k*_B_ the Boltzmann constant, *T*_h_ a characteristic temperature, *q* the elementary charge, *E* the electric field, and *r* the hopping distance. The electric field is a function of the voltages applied to the electrodes, *V*_SD_ and *V*_G_. For qualitative deduction, we rewrite Eq. (s1) as

$I_{D}=G_{h}e^{-\left( \frac{{k_{B}T}_{h}}{q(\epsilon_{1}V_{\mathrm{SD}}+\epsilon_{2}V_{G})r} \right)^{\frac{1}{3}}}$, (s2)

where $\epsilon_{1}$ and $\epsilon_{2}$ are generalized proportionalities. The transconductance is then

$$G_{m}=\frac{dI_{D}}{dV_{G}}=V_{\mathrm{SD}}G_{h}e^{-\left( \frac{{k_{B}T}_{h}}{q(\epsilon_{1}V_{\mathrm{SD}}+\epsilon_{2}V_{G})r} \right)^{\frac{1}{3}}}\frac{1}{3}\left( \frac{{k_{B}T}_{h}}{q(\epsilon_{1}V_{\mathrm{SD}}+\epsilon_{2}V_{G})r} \right)^{\frac{4}{3}}\frac{q\epsilon_{2}r}{{k_{B}T}_{h}}$$

$=I_{D}\frac{1}{3}\left( \frac{{k_{B}T}_{h}}{q(\epsilon_{1}V_{\mathrm{SD}}+\epsilon_{2}V_{G})r} \right)^{\frac{4}{3}}\frac{q\epsilon_{2}r}{{k_{B}T}_{h}}$. (s3)

Therefore, the signal induced by gate modulation is

$I_{\mathrm{sig}}= G_{m}V_{G}=V_{G}I_{D}\frac{1}{3}\left( \frac{{k_{B}T}_{h}}{q(\epsilon_{1}V_{\mathrm{SD}}+\epsilon_{2}V_{G})r} \right)^{\frac{4}{3}}\frac{q\epsilon_{2}r}{{k_{B}T}_{h}}$. (s4)

When the bias voltage $V_{\mathrm{SD}}$ increases, the drain current $I_{D}$ increases according to Equation s1. However, the corresponding signal $I_{\mathrm{sig}}$ does not increase linearly with $I_{D}$, because the rise of $I_{D}$ is accompanied by the rise of $V_{\mathrm{SD}}$, which leads to a decreasing term in between the brackets on the right side of Equation s4. That is to say, the proportionality of $I_{\mathrm{sig}}$ with respect to $I_{D}$ is decreasing when $I_{D}$ increases. Equation s4 shows why the signal scales with the drain current in a sublinear manner. Therefore, the signal power, defined as ${{P_{\mathrm{sig}}=I}_{\mathrm{sig}}}^{2}$ (see Experimental section), also scales sublinearly with ${I_{D}}^{2}$.

Equation s4 also indicates that the output signal $I_{\mathrm{sig}}$ is approximately proportional to $V_{G}$, when $\epsilon_{2}V_{G}$ is much smaller than $\epsilon_{1}V_{\mathrm{SD}}$, which is the case when the gate electrode is far away from the hopping path and the gate voltage is small. Therefore, the gate modulation of 0.1 V amplitude used for SNR characterization (see Figure 3 in the main text) is representative for the linear regime. Scaling this amplitude will result in a scaling of the SNR curve in the vertical direction, but no offset in the horizontal direction.

**Repeatability of results:**

To confirm the generality of our findings, we collected two more datasets. In one dataset (Figure S3), we switched the polarity of the source and drain electrodes of the boron dopant network device reported in the main text. In another dataset (Figure S4), we measured an arsenic dopant network device. The 1/*f* noise, the SNR peak, and the co-occurrence of rising edge of SNR and falling edge of the intelligence index are all reproduced.


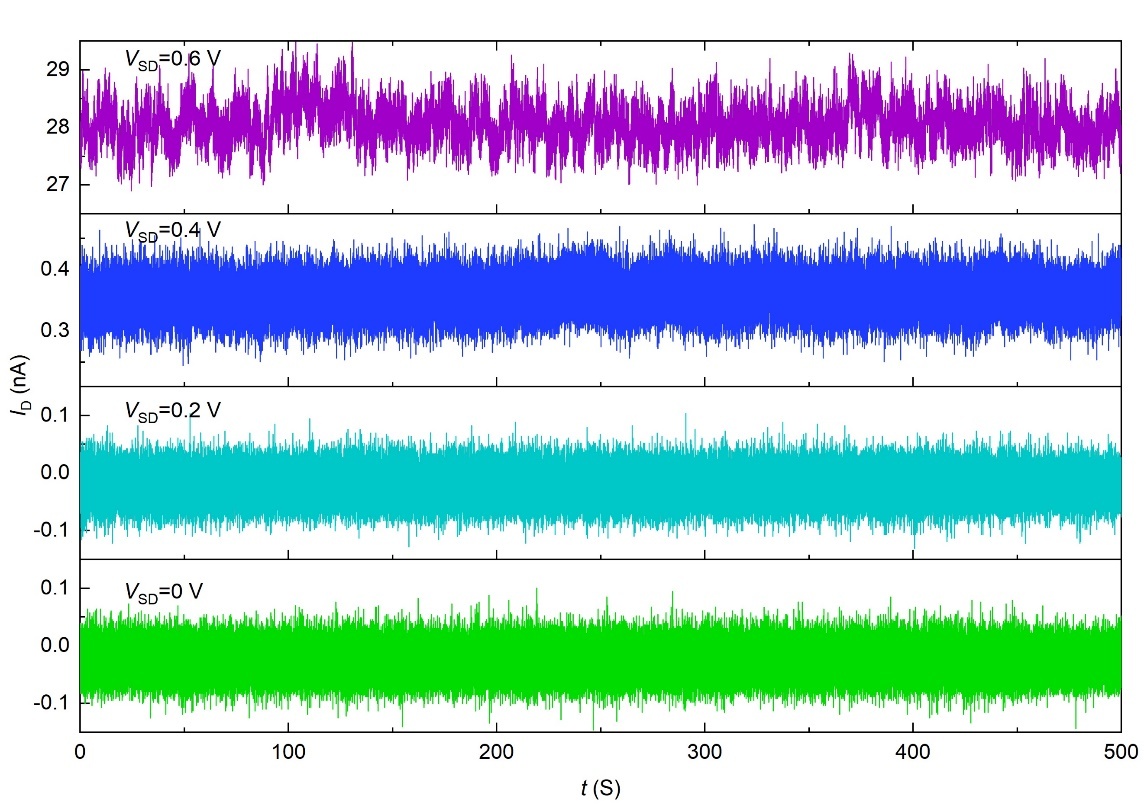


Figure S1. Drain current traces. The full length of 500 s of the drain current traces at different bias voltages have been plotted, which correspond to Figure 2A. The characteristics of the waveforms stay the same over the recording time, suggesting stationary states of the electron hopping dynamics when driven steadily by the bias voltage.


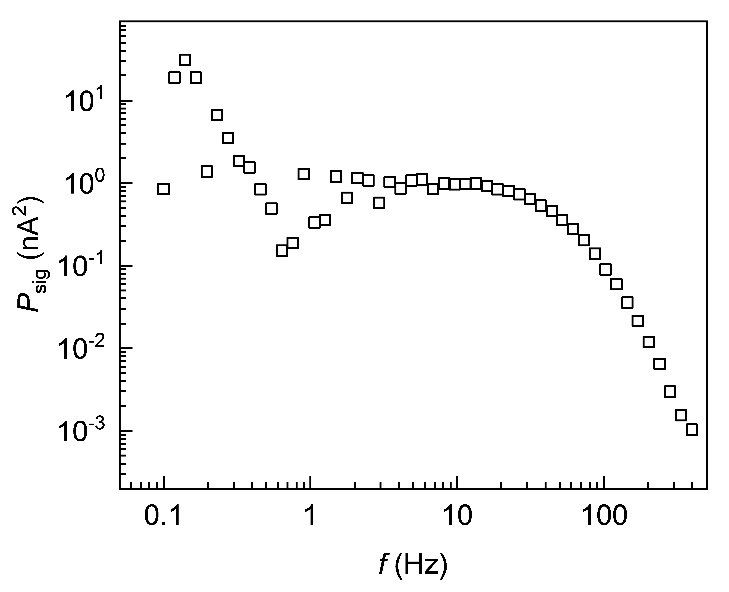


Figure S2. Bandwidth of the measurement setup. The roll-off at the high frequency end matches the roll-off in the top panel of Figure 2B (guided by short dashed line). The bandwidth is around 70 Hz, which agrees with the settings described in the Experimental section, i.e. a 200 Hz low-pass filter followed by a 100 Hz low-pass filter. The fluctuation of the signal intensity below 4 Hz is attributed to 1/f noise.


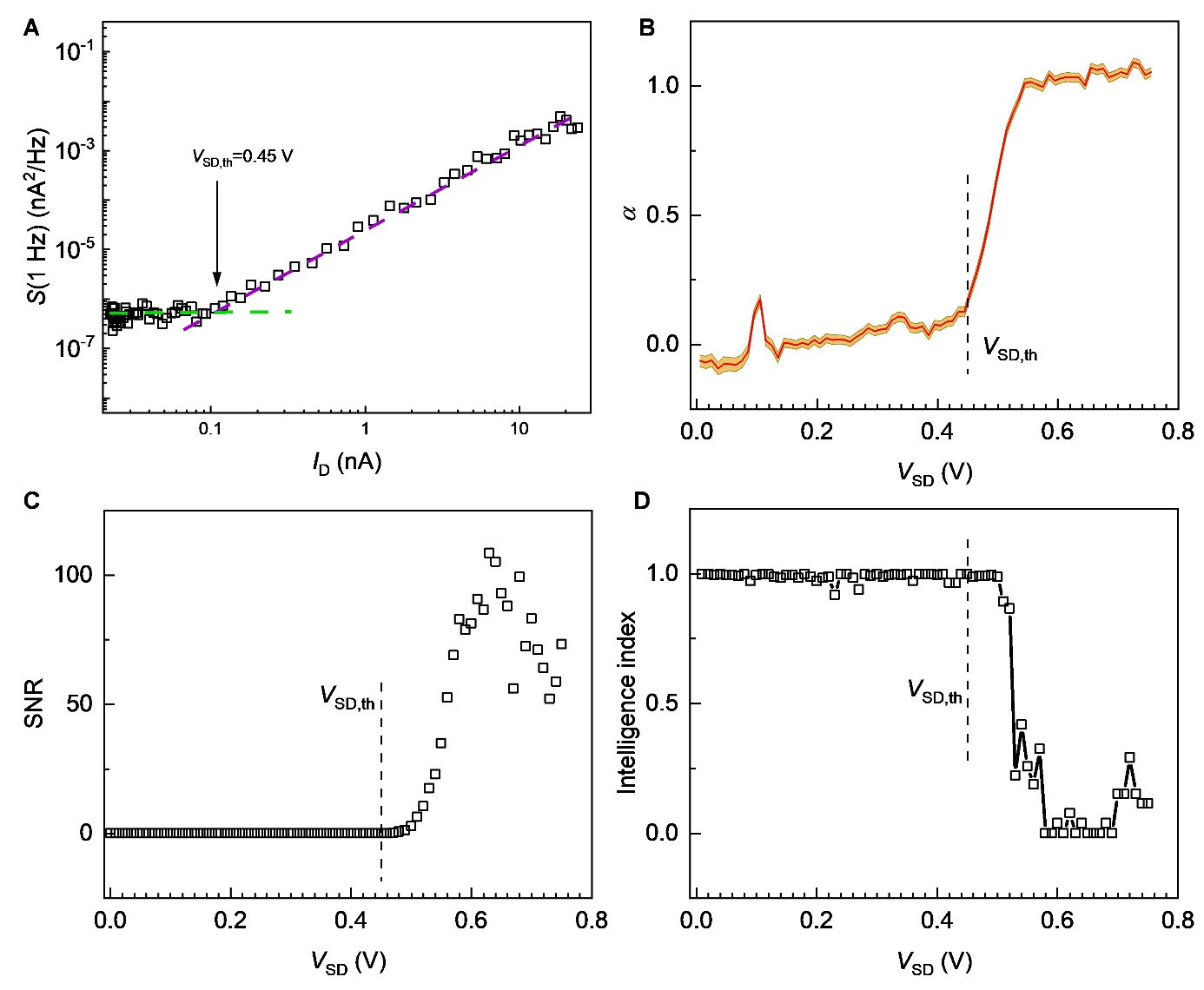


Figure S3. Experimental results with switched polarity of source and drain of the boron dopant network device reported in the main text. A, The threshold voltage is again determined by plotting the noise intensity at 1 Hz as a function of drain current. B, The exponent α rises to around 1 when the bias voltage exceeds V_SD,th_. We note that the threshold voltage here is different from that in the main text when the polarity is switched, because the electron hopping rate between two sites is not the same when source and destination sites are interchanged. C, SNR under gate modulation of 0.1 V and 1 Hz as a function of the bias voltage. The characteristic is the same as Figure 3, showing a peak around 0.64 V. D, Intelligence index as a function of bias voltage. The falling edge is clearly aligned with the rising edge of SNR, confirming the observation in the main text.

#
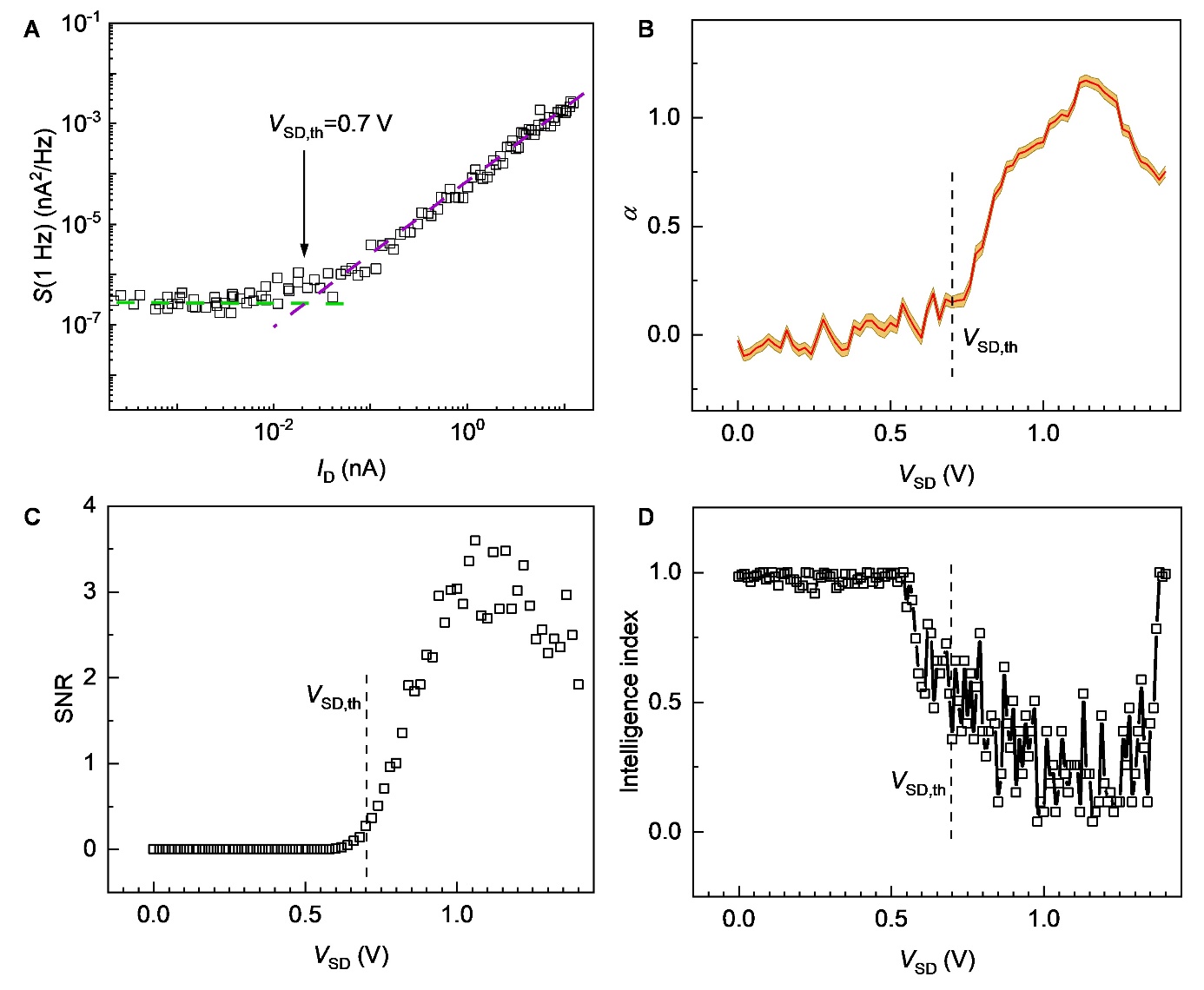


Figure S4. Experimental results of an arsenic dopant network device. A, The threshold voltage is determined by plotting the noise intensity at 1 Hz as a function of the drain current. B, The exponent α rises to a range of 0.74 to 1.2 when the bias voltage exceeds V_SD,th_. The deviation from 1 is attributed to finite-size effects of the dopant network.^[36]^. C, SNR under a gate modulation of 0.1 V and 1 Hz as a function of bias voltage. The characteristic is the same as in Figure 3. D, Intelligence index as a function of bias voltage. Although the measured arsenic device is less conductive than the boron device, all observations reported in the main text are reproduced.


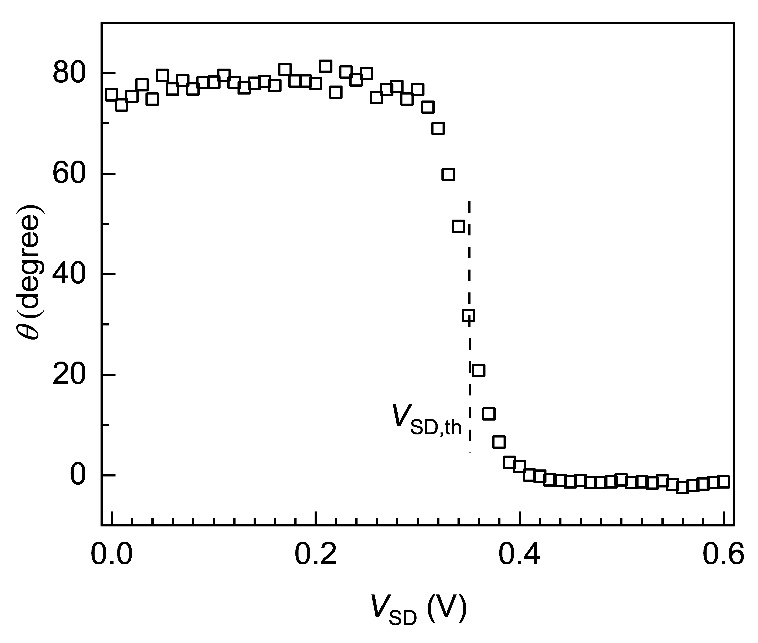


Figure S5. Phase of the response signal induced by the 1 Hz sine wave applied to the gate electrode. The falling edge matches V_SD,th_, above which the response signal through the dopant network is predominantly due to the gate-modulated hopping conduction. Below V_SD,th,_ the response signal at 1 Hz is attributed to cross coupling between wires of measurement setup.
